# High Dynamic Velocity Range Particle Image Velocimetry Using Multiple Pulse Separation Imaging

**DOI:** 10.3390/s110100001

**Published:** 2010-12-23

**Authors:** Tim Persoons, Tadhg S. O’Donovan

**Affiliations:** 1 Mechanical Engineering Department, Parsons Building, Trinity College, Dublin 2, Ireland; 2 School of Mechanical Engineering, Purdue University, 585 Purdue Mall, West Lafayette, IN 47907, USA; 3 School of Engineering and Physical Sciences, Nasmyth Building, Heriot-Watt University, Edinburgh EH14 4AS, UK; E-Mail: tso1@hw.ac.uk

**Keywords:** high dynamic range, velocity measurements, PIV, uncertainty analysis, impinging jet flow, laser-Doppler velocimetry, PACS 47.80.Cb

## Abstract

The dynamic velocity range of particle image velocimetry (PIV) is determined by the maximum and minimum resolvable particle displacement. Various techniques have extended the dynamic range, however flows with a wide velocity range (e.g., impinging jets) still challenge PIV algorithms. A new technique is presented to increase the dynamic velocity range by over an order of magnitude. The multiple pulse separation (MPS) technique (i) records series of double-frame exposures with different pulse separations, (ii) processes the fields using conventional multi-grid algorithms, and (iii) yields a composite velocity field with a locally optimized pulse separation. A robust criterion determines the local optimum pulse separation, accounting for correlation strength and measurement uncertainty. Validation experiments are performed in an impinging jet flow, using laser-Doppler velocimetry as reference measurement. The precision of mean flow and turbulence quantities is significantly improved compared to conventional PIV, due to the increase in dynamic range. In a wide range of applications, MPS PIV is a robust approach to increase the dynamic velocity range without restricting the vector evaluation methods.

## 1. Introduction

In particle image velocimetry (PIV), a flow is seeded with tracer particles and illuminated by a pulsed light sheet, yielding a series of image pairs with a pulse separation *τ*. After subdividing the images into interrogation windows, spatial cross-correlation yields the window-averaged particle displacement. The general theory and design rules for PIV have been established by e.g., Keane and Adrian [1,2]. Since the early 1990s, progressive improvements have been made to velocity evaluation methods. Some key contributions are reviewed below in terms of their influence on the dynamic velocity range DR*_V_*, corresponding to the ratio of maximum to minimum resolvable velocity:

(1) $${\text{DR}}_{V}=\frac{U_{\max}}{\sigma_{V}}=\frac{s_{\max}}{\sigma_{s}}$$

where *σ_V_* and *σ_s_* are the minimum resolvable velocity and displacement, respectively (*σ_V_* = *Mσ_s_*/*τ*). *σ_s_* is defined as $\sigma_{s}=\sqrt{\Delta s_{\mathrm{bias}}^{2}+\Delta s_{\mathrm{rms}}^{2}}{|}_{\hat{s}\to 0}$, where the bias error Δ*s_bias_* is the deviation between the true and measured displacement $s=\frac{1}{n}\sum_{1}^{n}s_{j}$, and the uncertainty $\Delta s_{\mathrm{rms}}=\sqrt{\frac{1}{n}\sum_{j=1}^{n}{\left(s_{j}-s\right)}^{2}}$.

### 1.1. Single-Pass Correlation

To avoid loss of correlation due to excessive in-plane displacement, Keane and Adrian [1] state that the displacement *s* should be smaller than one quarter of the interrogation window size *d_I_*, or $s=\tau U/M<\frac{1}{4}d_{I}$. This yields a maximum value for the pulse separation *τ* for a given velocity magnitude *U* and pixel scaling *M* [m/px]. Smaller *τ* values result in a slightly stronger correlation; however the displacement should remain greater than the minimum resolvable displacement. Incorporating this rule, the dynamic velocity range for single-pass correlation is

(2) $${\text{DR}}_{V}^{\left(s\right)}=\frac{\frac{1}{4}d_{I}}{\sigma_{s}^{\left(s\right)}}$$

Raffel *et al*. [3] and Westerweel [4] review the dependence of the total displacement error (and thus *σ_s_*) on a number of parameters for single-pass correlation (e.g., particle displacement, number density and diameter, interrogation window size, image background noise, velocity gradients).

### 1.2. Multi-Pass Correlation

Westerweel *et al*. [5] describe a multi-pass correlation approach by shifting windows over discrete pixel amounts, based on the local displacement obtained in the previous pass. Simulation results show a threefold reduction in displacement uncertainty Δ*s_rms_*. Validation results of grid-generated turbulence in a water channel show a typical displacement uncertainty of 0.04 px, compared to 0.095 px without window shifting [5]. The technique has since been improved to continuous shifting, applying image interpolation techniques [6].

Scarano and Riethmuller [7] describe an iterative window deformation method with progressive grid refinement. Monte Carlo simulations of noiseless artificial images yield uncertainty values of about 10^−3^ px [8]. Multi-grid techniques partly decouple the maximum displacement and final window size, since the 1/4 window rule [1] only applies to the first (coarse) grid. For a progressive refinement from an initial window *k_g_d_I_* to final window *d_I_* (*k_g_* > 1), Equation (2) can be rewritten as:

(3) $${\text{DR}}_{V}^{\left(m\right)}=k_{g}\frac{\frac{1}{4}d_{I}}{\sigma_{s}^{\left(m\right)}}$$

For the same final window (*d_I_*), DR*_V_* increases by the grid refinement ratio (typically 2 ≤ *k_g_* ≤ 4). A further increase is due to a reduction of *σ_s_*. Westerweel [5] and Scarano and Riethmuller [6] report an uncertainty reduction *σ_s_*^(s)^/*σ_s_*^(m)^ ≅ 3 for discrete window shifting and *σ_s_*^(s)^/*σ_s_*^(m)^ ≅ 10 for subpixel window shifting and deformation, respectively. However, these values are obtained for noiseless artificial images and the uncertainty increases for more realistic conditions, e.g., non-zero gradients [9].

In the remainder of the paper, ‘conventional’ PIV refers to the current state of art multi-grid cross-correlation using subpixel window shifting and deformation.

### 1.3. Multi-Frame (MF) Correlation: Locally Increasing Pulse Separation

Increasing the pulse separation to enhance the dynamic range is generally not preferred. However some studies present satisfactory results when the increase is applied locally [10–12]. These techniques use single-frame imaging, and are proposed as alternatives to multi-grid methods.

Fincham and Delerce [10] suggest a multi-frame (MF) approach based on a series of single-frame recordings, where an initial correlation of two frames (separated by inter-frame time *δt*) is used as a displacement estimate for the deformation and correlation of frames separated by 2*δt* or 3*δt*. This aim is to increase the average pixel displacement, thus improving the dynamic velocity range.

Hain and Kähler [11] also propose an iterative MF technique to compensate for the loss in dynamic range of CMOS sensors used in high speed PIV systems, compared to CCD sensors. On a single-frame sequence {...*t −* 2*δt*, *t − δt*, *t*, *t + δt*, *t +* 2*δt*...}, an initial correlation is performed on frames *t − δt* and *t + δt*. The correlation is repeated between frames *t − k_τ_δt* and *t + k_τ_δt*, where the multiplier *k_τ_* is estimated based on the quarter window rule assumption and the local displacement *s*, as $k_{\tau }=\frac{1}{4}d_{I}/s$. In selecting the optimal *k_τ_*, Hain and Kähler [11] indicate that a simple threshold for the correlation peak ratio *Q* (*i.e.*, ratio of highest to second highest correlation peak [1]) is not sufficient for optimality. The authors assume a minimum resolvable displacement of 0.1 px.

Multi-frame PIV is most suitable for low speed flows. Hain and Kähler [11] validate their technique with direct numerical simulations of a laminar separation bubble (*U_max_* = 0.15 m/s) and experimental velocity data around an airfoil in water (*U_max_* = 0.1 m/s). Pereira *et al*. [12] propose a similar MF technique and compare it to multi-grid PIV, for test cases including artificial particle images (*U_max_* = 1 px/s and *δt* = 1 s) and a laminar water flow (*U_max_* = 0.05 m/s). In these cases with a wide velocity range, MF PIV has achieved good results compared to conventional PIV. However, since MF PIV is proposed as an alternative to multi-grid algorithms, it cannot benefit from advances in this field.

### 1.4. Objectives

This paper proposes a new multiple pulse separation (MPS) technique to increase the dynamic velocity range of PIV. The technique is based on double-frame imaging, thus avoiding the low speed restriction and excessive pulse separations of MF PIV [10–12]. It does not exclude the use of multi-grid algorithms. A robust criterion for pulse separation optimality is established and validated.

## 2. Proposed Methodology: Multiple Pulse Separation (MPS) PIV

### 2.1. Basics of MPS PIV

Consider a flow field with a wide range in velocity magnitude (e.g., a jet or wake flow), where *U_max_* and *U_min_* represent two characteristic velocity scales in the high and low velocity regions, respectively. As the ratio *U_max_*/*U_min_* approaches the dynamic velocity range of the measurement technique (DR*_V_*), the vector quality in the low velocity region deteriorates. For this reason multi-frame correlation was first proposed [10–12]. By selectively applying a higher pulse separation *k_τ_τ* only in the low velocity region, the minimum measurable velocity reduces (*σ_V_* ∝ *σ_s_*/(*k_τ_τ*)) and the dynamic velocity range increases:

(4) $${\text{DR}}_{V}^{\left(\text{mps}\right)}=k_{g}\frac{\frac{1}{4}d_{I}\tau }{\sigma_{s}{}^{\left(m\right)}/\left(k_{\tau }\tau \right)}=\underset{\begin{array}{c} \text{pulse}\;\text{separation}\\ \text{multiplier} \end{array}}{\underset{︸}{k_{\tau }}}\underset{\begin{array}{c} \text{grid}\\ \text{refinement} \end{array}}{\underset{︸}{k_{g}}}\frac{\frac{1}{4}d_{I}}{\sigma_{s}{}^{\left(m\right)}}$$

The increase in DR*_V_* is proportional to the applied pulse separation multiplier *k_τ_*, which in turn is determined by the optimality criterion described in the following section.

**Figure 1.**
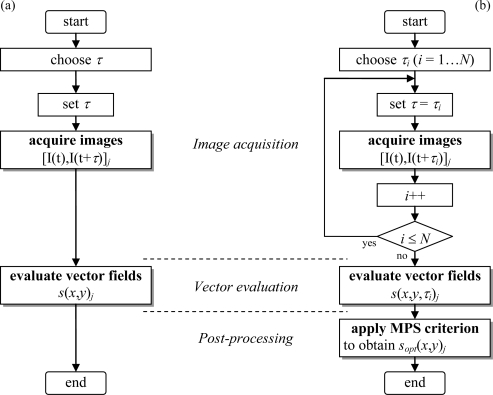
Flowchart for **(a)** conventional PIV and **(b)** multi pulse separation (MPS) PIV with optimal pulse separation criterion defined by Equation (9).

Contrary to the multi-frame approach [10–12], multi pulse separation (MPS) PIV acquires double-frame images {...,[*t*, *t + k_τ_*_,1_*τ*], [*t + δt*, *t + δt + k_τ_*_,2_*τ*],...} with *N* different pulse separation values *k_τ,i_τ* (*i* = 1…*N*) at a frame rate 1/*δt*, where the *N* multipliers *k_τ,_*_1_, *k_τ,_*_2_, … *k_τ,N_* represent monotonically increasing values (e.g., 1, 4, 16). Figure 1(a) depicts the conventional double-frame (single exposure) PIV approach with a single fixed pulse separation *τ*. The subscript *j* is the index in the sequence of acquired image pairs, and the subsequently evaluated displacement fields *s⃗* (*x,y*)*_j_* the arrow notation is often omitted hereafter). Figure 1(b) depicts the MPS PIV approach: (i) a sequence of double-frame images [I(*t*), I(*t*+*τ_i_*)]*_j_* is acquired, while the pulse separation loops through *N* chosen values (*τ_i_* = *k_τ,i_τ*). Next (ii) the vector fields for all pulse separation values are evaluated using conventional multi-grid algorithms. Finally (iii) the pulse separation optimality criterion (described below) is applied in a post-processing step, resulting in the final displacement fields *s⃗_opt_*(*x*,*y*)*_j_*.

### 2.2. Optimality Criterion for Pulse Separation

The peak ratio *Q* is a measure of the correlation strength of a displacement vector [1]. To assess the local precision, the displacement magnitude |*s*| is compared to the minimum resolvable displacement *σ_s_*. As a precision measure, 1 − *σ_s_*/|*s*| varies between unity for |*s*| *σ_s_*, over zero for |*s*| = *σ_s_* to -∞ as |*s*| → 0. The weighted peak ratio *Q’* is defined as a measure of local vector quality, combining correlation strength and precision:

(5) $$Q^{\prime }=Q\left(1-\frac{\sigma_{s}}{\left\|\vec{s}\right\|}\right)$$

The pulse separation optimality criterion is based on the local maximum of *Q’*. In each point (*x*, *y*), the local maximum of *Q’* = *Q’*(*x*,*y*,τ*_i_*) = *Q*(*x*,*y*,τ*_i_*) (1 − σ*_s_*/‖s⃗(*x*,*y*,τ*_i_*)‖) the local optimum pulse separation. The approach assumes that the value of *σ_s_* does not vary significantly within the field of view, which is true for typical laboratory conditions with background image noise and velocity gradients. Although advanced multi-grid algorithms can attain errors below 0.001 px in noiseless conditions [6,8], a value for *σ_s_* in more realistic conditions is about 0.1 px. In the optimality criterion, values of *σ_s_* between 0.05 px and 0.2 px yield the best results. This order of magnitude seems appropriate for multi-grid algorithms in realistic conditions, based on validation results in the review of Stanislas *et al*. [13].

A selector operator is defined based on the maximum *Q’* value:

(6) $$\begin{array}{l} \text{for}\;\text{any}\;\text{variable}\;a_{i}\left(\tau_{i}\right):\\ {\text{sel}}_{Q^{\prime }}\left(a_{i}\right)={a_{i}|}_{Q_{i}^{\prime }=\underset{i}{\text{max}}\left(Q_{i}^{\prime }\right)} \end{array}$$

The optimal pulse separation, displacement and velocity fields are determined as

(7) $$\begin{array}{l} \tau_{\mathrm{opt}}(x,y)={\text{sel}}_{Q^{\prime }}\left(\tau_{i}\right)={\tau_{i}|}_{Q_{i}^{\prime }=\underset{i}{\text{max}}\left(Q_{i}^{\prime }\right)}\\ {\vec{s}}_{\mathrm{opt}}(x,y)={\text{sel}}_{Q^{\prime }}\left(\vec{s}\left(x,y,\tau_{i}\right)\right)\\ {\vec{U}}_{\mathrm{opt}}(x,y)=\frac{M\;{\vec{s}}_{\mathrm{opt}}(x,y)}{\tau_{\mathrm{opt}}(x,y)} \end{array}$$

Based on Equation (7), each vector is taken from a single measurement according to the local maximum *Q’* value. An alternative definition is based on a linear combination, weighted according to the value of *Q’*. A relaxed maximum selector is therefore defined as

(8) $$\begin{array}{c} \text{for}\;\text{any}\;\text{variable}\;a_{i}\left(\tau_{i}\right):\\ {\text{sel}}_{Q^{\prime }}^{\left(p\right)}\left(a_{i}\right)=\frac{\sum a_{i}w_{i}}{\sum w_{i}}\text{with}\;w_{i}={\left[\frac{Q_{i}^{\prime }-\underset{i}{\text{min}}\left(Q_{i}^{\prime }\right)}{\underset{i}{\text{max}}\left(Q_{i}^{\prime }\right)-\underset{i}{\text{min}}\left(Q_{i}^{\prime }\right)}\right]}^{p} \end{array}$$

where *p* > 1. As *p* → ∞, the weights tend to *w_i_* = 1 for *Q′_i_* = max*_i_* *Q′_i_* and *w_i_* = 0 otherwise, and the relaxed maximum selector reverts to Equation (6), or $\underset{p\to \infty }{\text{lim}}\left({\text{sel}}_{Q^{\prime }}^{\left(p\right)}\left(a_{i}\right)\right)\equiv {\text{sel}}_{Q^{\prime }}\left(a_{i}\right)$.

Using Equation (8) the optimal displacement, velocity and pulse separation are

(9) $$\begin{array}{c} {\vec{s}}_{opt}(x,y)={\text{sel}}_{Q^{\prime }}^{\left(p\right)}\left[\vec{s}\left(x,y,\tau_{i}\right)\right]\\ {\vec{U}}_{opt}(x,y)={\text{sel}}_{Q^{\prime }}^{\left(p\right)}\left[\vec{U}\left(x,y,\tau_{i}\right)\right]\\ \tau_{opt}(x,y)=\frac{M\left\|{\vec{s}}_{opt}(x,y)\right\|}{\left\|{\vec{U}}_{opt}(x,y)\right\|} \end{array}$$

The optimality criterion based on the relaxed maximum (Equation (9)) yields smoother results since data obtained at different pulse separations are combined, weighted by the local *Q’* value. The exponent *p* determines the relative contribution of data obtained at sub-optimal pulse separations. In practice *p* = 5 yields good results, while the difference between Equations (7) and (9) is negligible for *p* > 20.

In choosing the pulse separation multipliers *k_τ,i_* (*i* = 1…*N*), the smallest value *τ* (*k_τ,_*_1_ = 1) should limit the correlation loss in the high velocity region, based on e.g., the 1/4 window rule [1] or similar considerations. The maximum *k_τ,N_* can be chosen analogously for the low velocity region, e.g., as. k_*τ,N*_ ≅ *U_max_*/*U_min_* Regarding the total number of values, *N* = 2 or 3 typically yields good results while limiting the additional acquisition and processing time.

Compared to conventional PIV, the maximum increase in dynamic velocity range is ${\text{DR}}_{V}^{\left(\text{mps}\right)}/{\text{DR}}_{V}^{\left(m\right)}=k_{\tau ,\text{max}}$ (see Equation (4)), where *k_τ,_*_max_ < *k_τ,N_* since the optimality criterion does not necessarily select the largest applied pulse separation. From Equation (4), the actual dynamic velocity range for MPS PIV is given by

(10) $${\text{DR}}_{V}^{\left(\text{mps}\right)}=k_{\tau }k_{g}\frac{\frac{1}{4}d_{I}}{\sigma_{s}^{\left(m\right)}}\text{with}\;k_{\tau }=\frac{\underset{x,y}{\text{max}}\left[\tau_{\mathrm{opt}}\left(x,y\right)\right]}{\tau }$$

where *τ_opt_*(*x*,*y*) follows from Equation (9). Depending on the flow conditions and the value of the minimum resolvable displacement *σ_s_*, the dynamic velocity range can increase by more than one order of magnitude compared to conventional multi-grid PIV, as shown in the validation results in Section 3.2.

### 2.3. Analogy to High Dynamic Range (HDR) Photography

Mann and Picard [14] introduced a technique to combine photographic images with different exposure times, to extend the dynamic intensity range beyond the restrictions of a digital sensor. A composite high dynamic range (HDR) image is generated as the weighted sum of all images. Weighting or ‘certainty’ functions are determined to favour mid-range intensity values, corresponding to the maximal sensor sensitivity and avoiding clipping near the edges of the range. Reinhard *et al*. [15] and Battiato *et al*. [16] discuss several weighting approaches to match the nonlinear response curve of an optical sensor array.

The MPS PIV technique proposed in this paper shows some analogies to HDR imaging. In both cases, a high dynamic range composite field is generated from a set of low dynamic range fields with different ‘exposure times’. Similarities persist in the optimality criterion used to construct the composite field. In HDR imaging, continuous weighting functions are used to provide a gradual transition between dark (underexposed) and bright (overexposed) regions. Thus each pixel contains information from all images in the set. MPS PIV also uses continuous weighting functions based on the local weighted peak ratio *Q’*, given by the relaxed maximum criterion (Equations (8) and (9)). However, a high exponent (*p* ≅ 5) is applied in Equation (8) to limit the contribution of data obtained at sub-optimal pulse separation values. In the extreme case where *p* → ∞, Equation (8) tends to Equation (6) and becomes a strict maximum selector, where each vector is selected from a single pulse separation acquisition.

The contribution of data from sub-optimal pulse separations should be limited in MPS PIV due to the strongly nonlinear nature of the correlation peak detection in PIV. Spurious vectors for excessive pulse separation values must not be allowed to propagate into the composite velocity field. As for any other technique, MPS PIV should be applied with good judgment.

### 2.4. Applicability and Limitations

Similar to conventional PIV, MPS PIV is applicable to stationary or non-stationary flows. MPS PIV correlates double-frame images separated by *τ_i_*, whereas MF PIV correlates single-frame images separated by multiples of δ*t*. Determined by the system repetition rate *f_F_*, the minimal δ*t* far exceeds the minimum pulse separation for double-pulsed systems (*τ* 1/*f_F_*_,_*_max_* *≤ δt*). This is a significant distinction between MPS and MF PIV. Excessive particle displacement limits MF PIV to low speed flows. Hain and Kähler [11] and Pereira *et al*. [12] report maximum velocities below 0.1 m/s in practical applications. Using double-frame imaging, MPS PIV is applicable to low and high speed flows in the same way as conventional PIV.

For temporal or spectral analyses, the common limitation for MPS and MF techniques is that a single recording duration *N*δ*t* (or *N*/*f_F_*) should be smaller than the flow time scale, where *N* is the number of pulse separations. The same restriction applies to conventional PIV, albeit for *N* = 1.

For amplitude domain analysis, no restrictions apply for single-point statistics (e.g., mean, variances and Reynolds stresses, higher order moments, probability density functions). For two-point statistics (e.g., spatial correlation) only point pairs acquired at the same measurement time should be considered.

MPS PIV is not an alternative but an addition to multi-grid techniques, without restricting the use of advanced methods such as window shifting and deformation. For the validation results (Section 3), the technique is implemented as a set of macro functions in LaVision Davis 7.2.2, using its multi-grid algorithms with deformation for vector evaluation.

## 3. Experimental Validation

The proposed methodology is validated based on experimental PIV data, obtained in an axisymmetric impinging jet. Two references are used for this validation: (i) Firstly, the precision of the mean and rms velocity is compared against laser-Doppler velocimetry (LDV). Secondly, the accuracy of the radial mass flux is verified against the mass conservation law.

### 3.1. Description of the Test Case

**Figure 2.**
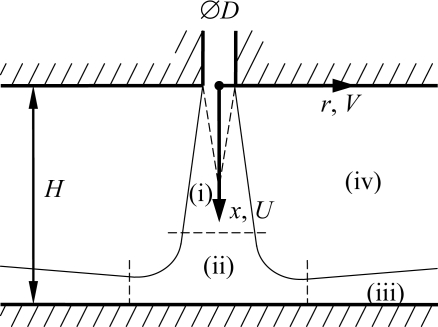
Description and nomenclature of the test case: Axisymmetric impinging jet flow.

A single round stationary jet of air impinges perpendicularly onto a flat surface (Figure 2). The orifice diameter *D* = 5 mm and the orifice-to-surface distance *H* = 4*D*. The axial and radial coordinates *x* and *r* are aligned along the jet axis and perpendicular to it, respectively. The jet issues from a straight-edged orifice of length 2*D*, connected to a settling chamber. The velocity distribution in the orifice is axisymmetric yet not radially uniform. This is not important for the test case, and no effort was made to prevent flow separation at the upstream orifice edge. The flow rate is measured and maintained constant using a digital mass flow controller (MKS 1579A, 300 standard litres/min, repeatability ±0.2%). All experiments are performed at a fixed Reynolds number of *Re* = 8,000, based on *D* and the mean velocity in the jet orifice (*U_m_* = 24 m/s). Fitzgerald and Garimella [17] present velocity distributions measured using LDV in a similar geometry for *Re* = 8,500.

Figure 2 identifies four distinct regions in the flow field: (i) the free jet with a decaying potential core in the centre and surrounding shear layer, (ii) the stagnation region, (iii) the wall jet and (iv) the entrainment region. Each of these features a significantly different characteristic velocity magnitude, making this an interesting test case for the proposed methodology.

The PIV system comprises a New Wave Solo-II Nd:YAG twin cavity laser (30 mJ, 15 Hz) and a LaVision FlowMaster 3S (PCO SensiCam) thermo-electrically cooled CCD camera (1,280 × 1,024 px^2^, 12 bit) with 28 mm lens. The image magnification is 1:3.4 (*M* = 45 μm/px). A glycol-water aerosol is used for seeding, with particle diameters between 0.2 and 0.3 μm. The particle image diameter is adjusted to *d_p_* ≅ 2 px by defocusing slightly. Customized optics generate a 0.3 mm thick light sheet. The CCD camera is mounted perpendicular to the light sheet. The velocity fields are processed with LaVision’s DaVis 7.2.2 software, using multi-grid cross-correlation with continuous window shifting and deformation, with a window size decreasing from 64 × 64 px^2^ to 32 × 32 px^2^ and a 75% overlap. The validation is based only on amplitude domain statistics (mean flow and turbulence intensities). As such, a low speed PIV system can be used in this stationary flow configuration.

The LDV system comprises a 500 mW Ar^+^ laser and a dual beam Dantec optics with 488 nm (blue) and 514 nm (green) wavelengths to measure axial (along *x*) and radial (along *r*) velocity components, respectively. The optical head applies Bragg cell frequency shifting to both components. The system is operated in backscattering mode to facilitate translation and near-wall measurements. The measurement volumes are about 0.12 mm in diameter and 1.6 mm long, with the long axis aligned in the out-of-plane (*z*) direction. The same aerosol seeding is used. The velocity data are evaluated using a Dantec BSA F50 burst spectrum analyser. Velocity weighting and statistics are performed using Matlab, applying inverse velocity magnitude weighting to reduce high velocity bias errors.

### 3.2. *Comparison of Conventional* versus *MPS PIV*

**Figure 3.**
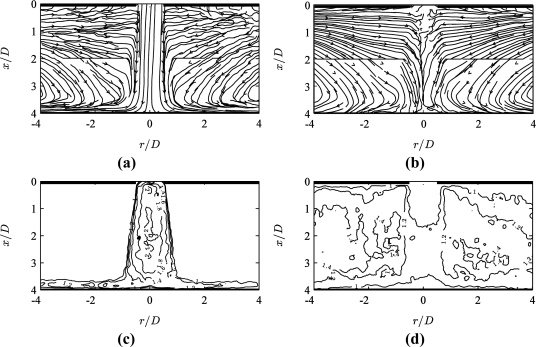
Conventional PIV results at pulse separation (a,c) *τ* = *τ_min_* (for resolving the high velocity jet region), and (b,d) *τ* = 10*τ_min_* (for resolving the low velocity entrainment region): **(a,b)** time-averaged streamlines and **(c,d)** corresponding weighted peak ratio *Q’*(*x*,*y*).

Figure 3(a) shows a time-averaged streamline plot for the jet flow obtained using conventional PIV. The term ‘conventional’ here denotes the best possible selection of the pulse separation *τ* = *τ_min_* which maximizes vector quality throughout the field of view, and the same above described algorithm. The quarter window rule in this case suggests $\tau <\frac{1}{4}Md_{I}/U_{\max}=30\;\mu s$, however a further reduction was needed due to strong gradients in the shear layer. To limit correlation loss due to gradients, Westerweel [9] derived a pulse separation threshold as $d_{I}|\partial U/\partial x|\tau <\frac{2}{3}d_{p}$ (for single pass correlation). For a shear layer gradient *∂U/∂x* ≅ *U_max_*/(D/2) = 9,600 s^−1^, the threshold yields *τ* < 4.3 μs. In practice, a maximum value of *τ* (= *τ_min_*) = 5 μs was found to ensure good vectors in the shear layer, resulting in a displacement of about 3 px in the jet core, and a gradient of *ds/dr*(*d_I_*/*d_p_*) ≅ 0.75 px/px in the shear layer. The strong gradient is the limiting factor here, yet the value of 0.75 px/px is comparable to that achieved by other authors in strong shear flows using multi-grid correlation [8]. Attempts to further increase *τ* (e.g., by decreasing the initial window) resulted in invalid vectors in the shear layer region.

Figure 3(b) shows the corresponding results for a 10 times larger pulse separation *τ* = 10*τ_min_*, yet otherwise identical acquisition and processing parameters. In the high velocity jet core region, the streamlines break down due to the absence of valid vectors, whereas the low velocity region shows smoother streamlines for *τ* = 10*τ_min_* than the ones for *τ* = *τ_min_* in Figure 3(a).

The MPS technique proposes the weighted peak ratio *Q’* = *Q*(1 – *σ_s_*/|*s*|) as a measure of local pulse separation optimality. Distributions of *Q’* are plotted in Figure 3(c,d). For both pulse separation values, the region of best vector quality corresponds to high values of *Q’*. These occur in the jet core and wall jet region for small pulse separation *τ* = *τ_min_* (Figure 3(a,c)) and in the entrainment region for the larger pulse separation *τ* = 10*τ_min_* (Figure 3(b,d)).

**Figure 4.**
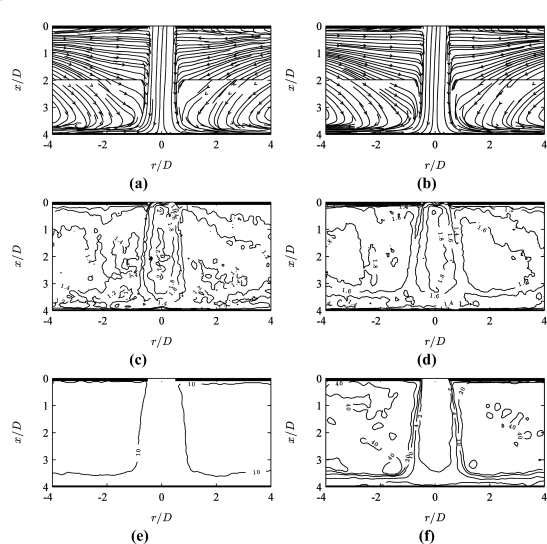
MPS PIV results (*σ_s_* = 0.2 px and *p* = 5 in Equation (8)) for data acquired at (a,c,e) *τ*/*τ_min_* = {1, 10} and (b,d,f) *τ*/*τ_min_* = {1, 2, 4, 10, 20, 40, 100}: **(a,b)** time-averaged streamlines, **(c,d)** weighted peak ratio *Q’*(*x*,*y*) and **(e,f)** local optimal pulse separation *τ_opt_*(*x*,*y*)/*τ_min_*.

Figure 4(a,c,e) shows the corresponding MPS PIV results after applying the optimality criterion (*σ_s_* = 0.2 px and *p* = 5 in Equation (8)) to the data obtained at two pulse separation values *τ*/*τ_min_* = {1, 10}. Figure 4(b,d,f) shows MPS results for data acquired at seven values *τ*/*τ_min_* = {1, 2, 4, 10, 20, 40, 100}.

Figure 4(e,f) shows the distribution of the optimal pulse separation *τ_opt_*(*x*,*y*)/*τ_min_*. The smallest values *τ* ≅ *τ_min_* are used in the jet core region, and larger values *τ* ≅ 10*τ_min_* in the entrainment region. When applying a larger number of pulse separations, Figure 4(f) shows that intermediate values 2 < *τ*/*τ_min_* < 4 are used for the stagnation and wall jet regions, and high values 10 < *τ*/*τ_min_* < 40 in the lowest velocity regions.

#### 3.2.1. Effect of Optimality Criterion Parameters

**Figure 5.**
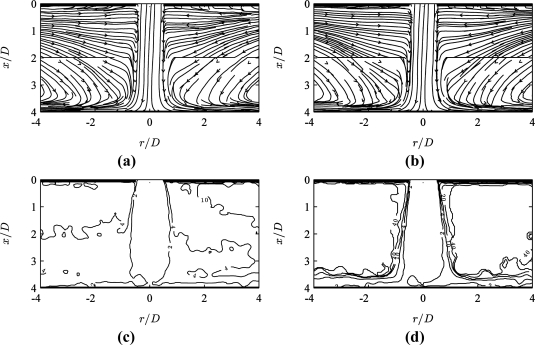
MPS PIV results with (a,c) *σ_s_* = 0.02 px and *p* = 5 (in Equation (8)) and (b,d) *σ_s_* = 0.2 px and *p* → +∞ for data acquired at *τ*/*τ_min_* = {1, 2, 4, 10, 20, 40, 100}: **(a,b)** time-averaged streamlines and **(c,d)** local optimal pulse separation *τ_opt_*(*x*,*y*)/*τ_min_*.

Figure 5 shows the influence of the optimality criterion parameters (*σ_s_* and *p* in Equation (8)) on the MPS PIV results. With a lower value of *σ_s_* (= 0.02 px), Figure 5(a,c) shows the criterion giving preference to high correlation strength rather than large pulse separation values, although a larger pulse separation (*τ* ≅ 10*τ_min_*) is still applied in the outer entrainment region. As *σ_s_* → 0 px, *Q’* → *Q* and thus the criterion selects the pulse separation corresponding to the maximum correlation peak ratio.

Figure 5(b,d) shows the effect of the strict maximum selector (Equation (6)), corresponding to *p* → +∞ in Equation (8). In this case, each vector is selected from a single pulse separation acquisition. The resulting distribution of the optimal pulse separation in Figure 5(d) shows discrete steps in pulse separation values applied throughout the flow field.

Comparing Figures 4 and 5, the effect of the criterion parameters on the streamline plot is not very significant. In that sense, the criterion is quite robust against parameter changes. However closer inspection of the results does allow optimisation of the criterion parameters *σ_s_* and *p*.

#### 3.2.2. Actual Increase of Dynamic Velocity Range

Based on Equation (10) and the results shown in Figure 4(f), the actual increase in dynamic velocity range can be determined. The ratio of local maximum to minimum pulse separation *k_τ_* = max(*τ_opt_*)/*τ_min_* ≅ 40. Therefore MPS has increased the dynamic velocity range by *k_τ_* ≅ 40 = 10^1.6^ times compared to the conventional multi-grid PIV approach. Determining the exact dynamic range based on Equation (10) is not straightforward. Assuming *σ_s_*^(m)^ ≅ 0.1 px and *k_g_d_I_* = 64 px, DR*_V_*^(m)^ ≅ 160:1 (= 10^2.2^). With this assumption, the dynamic range of MPS PIV is DR*_V_*^(mps)^ ≅ 6,400:1 (= 10^3.8^).

Although data was available at a higher pulse separation (*τ* = 100*τ_min_*), the optimality criterion has not used this, since the weighted peak ratio for *τ* = 100*τ_min_* is lower than for *τ* = 40*τ_min_* even in the low velocity region. This demonstrates that the technique does not necessarily select the largest pulse separation over the optimal value.

A dynamic velocity range of four orders of magnitude (10^4^:1) has already been quoted in the literature for multi-grid algorithms using a single pulse separation [6,8]. However, those values correspond to simulation results for noiseless artificial particle images, whereas this value of DR*_V_*^(mps)^ ≅ 6,400:1 (or 3.8 orders of magnitude) is obtained in laboratory conditions for a real jet flow.

### 3.3. Validation against Independent References

#### 3.3.1. Validation against Laser-Doppler Velocimetry

**Figure 6.**
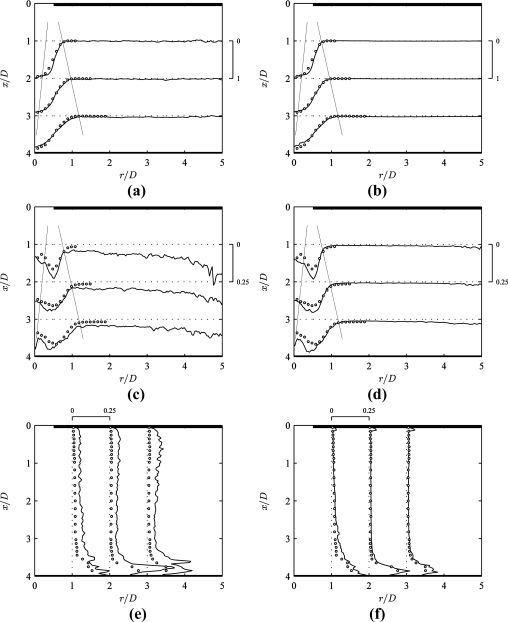
Comparison of (a,c,e) conventional PIV and (b,d,f) MPS PIV (*σ_s_* = 0.2 px and *p* = 5 in Equation (8)) against LDV measurements (circular markers): profiles of **(a,b)** time-averaged axial velocity *U*(*r*)/*U_m_*, **(c,d)** axial turbulence intensity *u’*(*r*)/*U_m_* and **(e,f)** radial turbulence intensity *v’*(*x*)/*U_m_*.

Figure 6 presents profiles of mean flow and turbulence intensity obtained using conventional (left) and MPS PIV (right) in the impinging jet. These quantities are defined as $U(=\bar{U})=\frac{1}{n}\sum_{j=1}^{n}U_{j}$ and $u^{\prime }=\sqrt{\frac{1}{n}\sum_{j=1}^{n}{\left(U_{j-}\bar{U}\right)}^{2}}$, where *U_j_* are the instantaneous velocity fields (*j* = 1…*n*) with analogous expressions for *V* and *v’*. All MPS PIV results hereafter correspond to the data in Figure 4(b) obtained at seven pulse separation values 1 ≤ *τ*/*τ_min_* ≤ 100, with *σ_s_* = 0.2 px and *p* = 5 in Equation (8). The circular markers represent measurements using the laser-Doppler velocimeter (LDV) described in Section 3.1. The extent of the jet core and outer shear layer is indicated by thin lines in Figure 6 (a–d). All velocities are normalised to the mean orifice velocity *U_m_* (= 24 m/s for *Re* = 8000).

In Figure 6(a,b), the time-averaged velocity results of conventional PIV, MPS PIV and LDV show a good agreement in the central region (*r*/*D* < 2) to within 5% deviation. The conventional PIV results exhibit some residual noise from averaging bad vectors in the low velocity region (*r*/*D* > 2), whereas the MPS PIV profiles are much smoother.

The difference is even clearer for the rms velocity fluctuations *u’* and *v’* (Figure 6(c–f)). The conventional PIV results only agree with LDV in the central region (*r*/*D* < 0.75) to within 5% (Figure 6(c)). However in the outer shear layer (*r*/*D* ≅ 1), conventional PIV overpredicts the turbulence intensity by about 2.5 times. In the entrainment region, conventional PIV falsely predicts a turbulence level of about 7.5% for 1.5 < *r*/*D* < 4, increasing up to 20% for *r*/*D* > 4. This behaviour has no physical ground, since LDV results by Fitzgerald and Garimella [17] confirm a turbulence intensity below 2% for *r*/*D* > 1.5 (for *Re* = 8500). This is verified in the MPS PIV turbulence intensity values of about 1.5% for 1.5 < *r*/*D* < 4 (Figure 6(d)).

Radial turbulence intensity profiles intersecting the wall jet region (Figure 6(e)) show an overprediction of about 7.5% for conventional PIV. Figure 6(f) shows a much better agreement in the wall jet region for MPS PIV, with an average deviation below 2%. The magnitude and location of the turbulence peak in the wall jet agrees well for MPS PIV and LDV results.

This validation against LDV shows that conventional PIV overestimates the turbulence intensity because the displacement magnitude reduces to the minimum resolvable level *σ_s_*, resulting in a poor velocity resolution. MPS PIV yields more precise results due to the increase in dynamic velocity range and reduction in minimum resolvable velocity (*σ_V_* ∝ min(*σ_s_*/*τ_opt_*)).

#### 3.3.2. Validation against Conservation of Mass

The increase in accuracy when applying MPS PIV can be quantified by verifying the conservation of mass in the flow field. For an axisymmetric impinging jet, the net mass flow rate *ṁ*(*r*) exiting a cylindrical control volume of radius *r* (see Figure 2) is given by:

(11) $$\dot{m}\left(r\right)=\rho \int_{x=0}^{H}2\pi rV\left(x,r\right)dx$$

This integral is obtained from the time-averaged velocity field, after averaging both half-planes for negative and positive *r* values (accounting for the reflection symmetry). Based on the conservation of mass, *ṁ*(*r*) should equal the jet flow rate *ṁ_jet_* for *r*/*D* > 0.5, where *ṁ_jet_* is determined by the mass flow controller measurement in the inlet duct. Since = *ṁ*(*r*)/*ṁ_jet_* = 1 represents the true value, the deviation of the PIV results allows to assess the increase in accuracy due to using the MPS technique.

**Figure 7.**
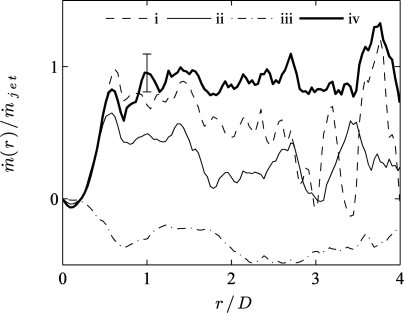
Radial profile of the mass flow rate *ṁ*(*r*)/*ṁ_jet_* for (i-iii) conventional PIV using a pulse separation (i) *τ* = *τ_min_*, (ii) *τ* = 4*τ_min_* and (iii) *τ* = 20*τ_min_*, compared to (iv) MPS PIV results for identical conditions as Figure 4(b,d,f) and Figure 6(b,d,f).

Figure 7 shows the radial profile of *ṁ*(*r*)/*ṁ_jet_*. The three thin lines (dashed, solid, dash-dotted) represent conventional PIV results at different pulse separations. The best agreement to *ṁ*(*r*)/*ṁ_jet_* = 1 is achieved for low *τ* values (cases (i) and (ii)), although the typical deviation exceeds 20% and the agreement breaks down for *r*/*D* > 1.5. As expected, the higher *τ* value (case (iii)) gives a very poor agreement due to bad vector quality, resulting from correlation loss in the jet shear layer and wall jet.

By contrast, the thick solid line (case (iv)) represents the mass flow rate for the MPS PIV flow field, which is the only result showing a reasonable agreement with *ṁ*(*r*)/*ṁ_jet_* = 1 for *r*/*D* > 0.5. The rms deviation of 5–7% is comparable in magnitude to the uncertainty on *ṁ_jet_*, obtained from the mass flow controller reading (2% based on the flow rate for *Re* = 8,000). The agreement holds quite well up to *r*/*D* < 3.5. This validation based on mass conservation provides quantifiable evidence for the higher accuracy achieved with MPS PIV compared to conventional PIV in this test case.

## 4. Conclusions

Multi pulse separation (MPS) PIV is presented as a new methodology to increase the dynamic velocity range of PIV, based on a combination of data obtained at multiple pulse separation values. The methodology applies to flow configurations with large variations in velocity magnitude within the field of interest, of the order of the dynamic velocity range.

The pulse separation optimality criterion is based on a weighted peak ratio defined as *Q’* = *Q*(1 – *σ_s_*/|*s*|), where the parameter *σ_s_* represents the minimum resolvable particle displacement. The optimised velocity field is obtained from Equations (8) and (9). Suitable values for *σ_s_* are between 0.05 px and 0.2 px, corresponding to the minimum resolvable displacement in typical laboratory conditions [13].

The MPS technique has been validated on an impinging jet flow, featuring strong velocity gradients and a wide range in velocity magnitude between the jet core, stagnation, wall jet and entrainment regions. Compared to laser-Doppler velocimetry (LDV) as a reference, conventional PIV significantly overpredicts the turbulence intensity by 7.5% (relative to *U_m_*) in the shear layer and wall jet, and up to 20% in the entrainment region. MPS PIV shows an excellent agreement to within 2% of the LDV results throughout the flow field.

The increase in dynamic velocity range also improves the accuracy, which is verified against the conservation of mass in a control volume around the impinging jet flow. An rms deviation below 7% is obtained using MPS PIV, compared to over 20% using conventional PIV.

The enhancement using MPS PIV in terms of accuracy and precision of mean flow and turbulence quantities is due to the significant increase in dynamic velocity range. Here, the actual dynamic velocity range has increased by 40 times, to 3.8 orders of magnitude (DR*_V_*^(mps)^ ≅ 6,400:1).

In other configurations with a wide velocity range, MPS has contributed to the understanding of heat transfer mechanisms e.g., in synthetic jet flows [18,19] and natural convection plumes around heated cylinders [20]. It could also enhance other PIV-based techniques, such as pressure field reconstruction [21]. MPS PIV is subject to similar limitations as conventional double-frame PIV in terms of temporal resolution (see Section 2.4). No restrictions are imposed on the vector evaluation method. The straightforward and robust method resolves strong gradients and a wide velocity range in a single recording sequence comprising multiple pulse separations. MPS PIV achieves order of magnitude enhancements of accuracy and precision of the mean and turbulent flow field, as proven by the validation results in this paper.

## Nomenclature

*D*: hydraulic diameter of the jet nozzle (m)
DR*_V_*: dynamic velocity range
*d_I_*: interrogation window size (px)
*d_p_*: particle image diameter (px)
*f_F_*: frame rate (Hz)
*H*: distance between the jet nozzle exit and impingement surface (m)
*k_g_*, *k_τ_*: grid refinement factor and pulse separation multiplier
*M*: image pixel scaling (m/px)
*ṁ*: mass flow rate (kg/s)
*n*: number of acquired image pairs
*N*: number of pulse separation values
*p*: exponent in relaxed maximum selector (see Equation (8))
*Q*, *Q’*: unweighted and weighted correlation peak ratio (*Q’* = *Q*(1 - *σ_s_*/|*s*|))
*Re*: jet Reynolds number, based on *D* and mean jet velocity
*r*: radial coordinate in impinging jet (m)
*s*: particle image displacement (px)
*U, V*: in-plane velocity (m/s)
*x*, *y*: in-plane coordinates (m)

**Greek symbols**
δ*t*: camera inter-frame time (frame rate = 1/δ*t*) (s)
Δ*s*: absolute displacement error or uncertainty (px)
*ρ*: fluid density (kg/m^3^)
*σ_s_*, *σ_V_*: minimum resolvable displacement (px) and velocity (m/s)
*τ*: pulse separation time between exposures (s)

**Subscripts**
*i*: index of pulse separation values
*j*: index of image pair in sequence
*rms*: uncertainty (*i.e.*, random error)
*bias*: bias (*i.e.*, systematic error)

**Superscripts**
(s): single-pass correlation
(m): multi-grid correlation
(mps): multiple pulse separation PIV

## References

[1] Keane R.D., Adrian R.J. (1990). Optimization of particle image velocimeters. Part I: Double pulsed systems. Meas. Sci. Technol.

[2] Keane R.D., Adrian R.J. (1992). Theory of cross-correlation analysis of PIV images. Appl. Sci. Res.

[3] Raffel M., Willert C., Kompenhans J., Adrian R.J., Gharib M., Merzkirch W., Rockwell D., Whitelaw J.H. (1998). Particle Image Velocimetry: A Practical Guide.

[4] Westerweel J. (1997). Fundamentals of digital particle image velocimetry. Meas. Sci. Technol.

[5] Westerweel J., Dabiri D., Gharib M. (1997). The effect of a discrete window offset on the accuracy of cross-correlation analysis of digital PIV recordings. Exp. Fluids.

[6] Scarano F., Riethmuller M.L. (2000). Advances in iterative multigrid PIV image processing. Exp. Fluids.

[7] Scarano F., Riethmuller M.L. (1999). Iterative multigrid approach in PIV image processing using discrete window offset. Exp. Fluids.

[8] Scarano F. (2002). Iterative image deformation methods in PIV. Meas. Sci. Technol.

[9] Westerweel J. (2008). On velocity gradients in PIV interrogation. Exp. Fluids.

[10] Fincham A., Delerce G. (2000). Advanced optimization of correlation imaging velocimetry algorithms. Exp. Fluids.

[11] Hain R., Kähler C.J. (2007). Fundamentals of multiframe particle image velocimetry (PIV). Exp. Fluids.

[12] Pereira F., Ciarravano A., Romano G.P., Di Felice F. Adaptive multi-frame PIV.

[13] Stanislas M., Okamoto K., Kähler C.J., Westerweel J. (2005). Main results of the second international PIV challenge. Exp. Fluids.

[14] Mann S., Picard R.W. On being ‘undigital’ with digital cameras: Extending dynamic range by combining differently exposed pictures.

[15] Reinhard E., Ward G., Pattanaik S., Debevec P. (2005). High Dynamic Range Imaging: Acquisition, Display, and Image-Based Lighting.

[16] Battiato S., Castorina A., Mancuso M. (2003). High dynamic range imaging for digital still camera: An overview. J. Electron. Imaging.

[17] Fitzgerald J.A., Garimella S.V. (1998). A study of the flow field of a confined and submerged impinging jet. Int. J. Heat Mass Transfer.

[18] Valiorgue P., Persoons T., McGuinn A., Murray D.B. (2009). Heat transfer mechanisms in an impinging synthetic jet for a small jet-to-surface spacing. Exp. Therm. Fluid Sci.

[19] Persoons T., Farrelly R., McGuinn A., Murray D.B. High dynamic range whole-field turbulence measurements in impinging synthetic jets for heat transfer applications.

[20] Persoons T., O Gorman I.M., Byrne G., Murray D.B. Time-resolved heat transfer and fluid dynamic analysis of natural convection from isothermal horizontal cylinders.

[21] Vanierschot M., van den Bulck E. (2008). Planar pressure field determination in the initial merging zone of an annular swirling jet based on stereo-PIV measurements. Sensors.

